# The interplay of serotonin 5-HT1A and 5-HT7 receptors in chronic stress

**DOI:** 10.1242/jcs.262219

**Published:** 2024-10-11

**Authors:** Monika Bijata, Alexander Wirth, Jakub Wlodarczyk, Evgeni Ponimaskin

**Affiliations:** ^1^ Nencki Institute of Experimental Biology, Polish Academy of Sciences, Pasteura 3, 02-093 Warsaw, Poland; ^2^ Cellular Neurophysiology, Center of Physiology, Hannover Medical School, Carl-Neuberg-Str. 1, 30625 Hannover, Germany

**Keywords:** Serotonin receptors, 5-HT7R, 5-HT1AR, Chronic stress, Heterodimerization

## Abstract

Serotonin regulates multiple physiological and pathological processes in the brain, including mood and cognition. The serotonin receptors 5-HT1AR (also known as HTR1A) and 5-HT7R (also known as HTR7) have emerged as key players in stress-related disorders, particularly depression. These receptors can form heterodimers, which influence their functions. Here, we explored the developmental dynamics of 5-HT1AR and 5-HT7R expression and validated heterodimerization levels in the brain of control and stressed mice. In control animals, we found that there was an increase in 5-HT1AR expression over 5-HT7R in the prefrontal cortex (PFC) and hippocampus during development. Using a chronic unpredictable stress as a depression model, we found an increase in 5-HT7R expression exclusively in the PFC of resilient animals, whereas no changes in 5-HT1AR expression between control and anhedonic mice were obtained. Quantitative *in situ* analysis of heterodimerization revealed the PFC as the region exhibiting the highest abundance of 5-HT1AR–5-HT7R heterodimers. More importantly, upon chronic stress, the amount of heterodimers was significantly reduced only in PFC of anhedonic mice, whereas it was not affected in resilient animals. These results suggest an important role of brain-region-specific 5-HT1AR–5-HT7R heterodimerization for establishing depressive-like behaviour and for development of resiliency.

## Teaser

**Summary:** There is a decreased level of 5-HT1AR–5-HT7R heterodimerization in the prefrontal cortex of anhedonic mice, suggesting that heterodimerization has a role in development of stress resiliency.

## INTRODUCTION

Serotonin (5-hydroxytryptamine, 5-HT) is a neurotransmitter that regulates various physiological and behavioural processes, such as mood, cognition, sleep and pain. Serotonin exerts its effects through binding to multiple receptors, which belong to seven families (5-HT1 to 5-HT7) and have different signalling and distribution properties (Nichols and Nichols, 2008; Sharp and Barnes, 2020). Among them, 5-HT1A (also known as HTR1A) and 5-HT7 (also known as HTR7) receptors have attracted considerable attention for their roles in mood disorders, such as depression and anxiety (Albert and Vahid-Ansari, 2019; Bijata et al., 2022a; Hedlund et al., 2005; Hirvonen et al., 2008; Lemonde et al., 2003).

5-HT1AR and 5-HT7R have different modulatory effects on the nervous system because they are coupled to G proteins that lead to opposite effects on signalling pathways. Activation of the 5-HT1AR causes activation of the Gαi protein, which results in a decrease in the activity of adenylate cyclase and, consequently, a decrease in the level of cyclic adenosine-5′-monophosphate (cAMP) (Albert and Robillard, 2002). In addition, 5-HT1AR activates the ERK1 and ERK2 (ERK1/2) mitogen-activated kinases (also known as MAPK3 and MAPK1, respectively) (Della Rocca et al., 1999). Moreover, 5-HT1AR stimulation leads to activation of G protein-coupled inwardly rectifying K^+^ channels (GIRK) via βγ subunits of G protein. This leads to membrane hyperpolarization, reduced neuronal excitability and inhibition of voltage-dependent Ca^2+^ channels, and consequently – reduced Ca^2+^ influx (Bayliss et al., 1997; Chen and Penington, 1996; Penington and Kelly, 1990; Penington et al., 1991). In contrast, activation of the 5-HT7R leads to activation of the Gαs protein, which increases the level of cAMP (Lovenberg et al., 1993; Tsou et al., 1994) resulting in increased depolarization of the cell membrane and neuronal excitability (Kusek et al., 2015). Therefore, the interplay between 5-HT1AR and 5-HT7R signalling might influence the cellular responses to serotonin (Renner et al., 2012). Previous studies have indicated crucial roles for 5-HT1A and 5-HT7R in neural development and stress responses (Albert and Vahid-Ansari, 2019; Bijata et al., 2017, 2022a; Gorinski et al., 2019; Guscott et al., 2005; Kobe et al., 2012; Tokarski et al., 2012a,b; Wesołowska et al., 2006a,b, 2007). However, a comprehensive understanding of their nuanced interplay and alterations during chronic stress still remains elusive (Kulikov et al., 2018; Naumenko et al., 2014).

We have previously demonstrated that 5-HT1AR and 5-HT7R can form homo- and hetero-dimers (Prasad et al., 2019; Renner et al., 2012). Heterodimerization attenuated the ability of 5-HT1AR to activate Gαi protein by 5-HT1AR without affecting 5 HT7R-related Gαs signalling. Moreover, 5-HT1AR–5-HT7R heterodimers have been shown to enhance serotonin-induced internalization of 5-HT1AR (Renner et al., 2012). Our data also suggested that the regulated ratio of serotonin receptors might be crucially involved in both the onset and response to treatments of psychiatric diseases, such as anxiety and depression.

In this study, we combined molecular, biochemical and imaging techniques to investigate the expression profile of 5-HT1AR and 5-HT7R and their heterodimers in the mouse brain under physiological conditions and after chronic unpredictable stress (CUS), a well-established animal model of depression (Antoniuk et al., 2019; Bijata et al., 2022b). To this end, we focused on three brain regions: the prefrontal cortex (PFC), the hippocampus (HP) and the raphe nuclei (RN). Among others, these regions are involved in the regulation of mood and stress response, and are implicated in the pathophysiology of depression (Dean and Keshavan, 2017; Ferrari and Villa, 2017; Palazidou, 2012). In control animals, we found a significant increase in mRNA levels for 5-HT1AR over the 5-HT7R at postnatal day (P)90 in PFC and HP, but not in RN. In addition, our study demonstrated that the expression of 5-HT7R was significantly increased in the PFC of resilient animals, whereas expression of 5-HT1AR was not affected. More importantly, the number of 5-HT1AR–5-HT7R heterodimers was significantly decreased in the PFC of anhedonic but not resilient mice subjected to the CUS protocol, suggesting a possible role of heterodimerization in development of resilient phenotype.

## RESULTS

### Expression dynamics of 5-HT7R and 5-HT1AR during the brain development

**Fig. 1.**
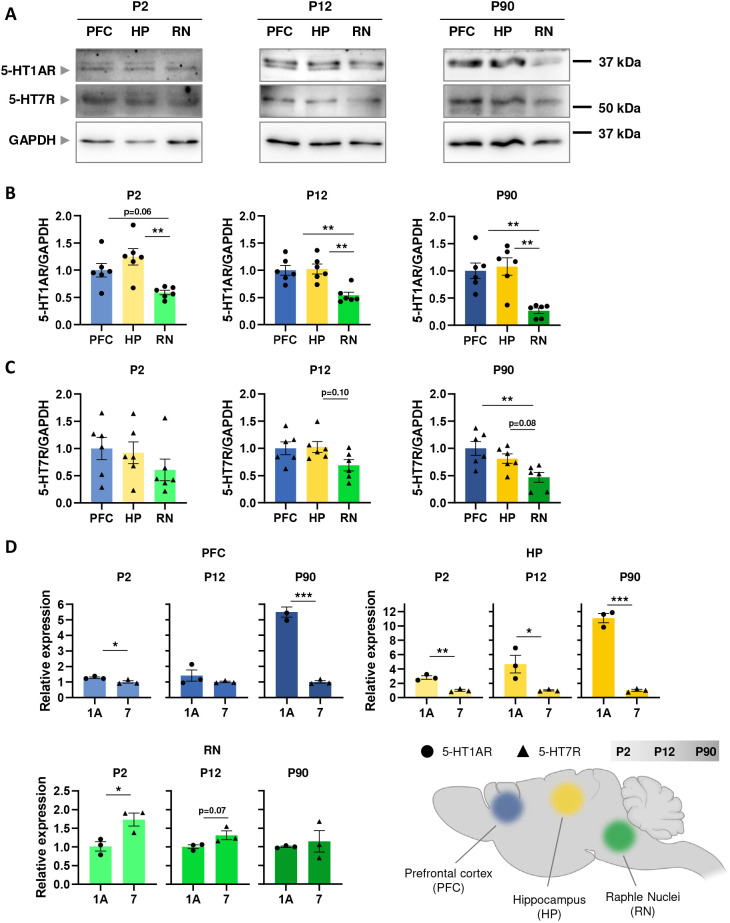
**Developmental dynamics of 5-HT1AR and 5-HT7R expression in the PFC, HP and RN.** (A) Representative western blot showing 5-HT1AR, 5-HT7R and GAPDH in the PFC, HP and RN at postnatal day 2 (P2), 12 (P12) and 90 (P90). (B,C) Quantification of the 5-HT1AR (B) and 5-HT7R (C) expression in HP, PFC and RN in P2, P12 and P90 (*n*_mice_=6). (D) Relative expression of mRNA encoding 5-HT1AR and 5-HT7R in PFC, HP and RN in P2, P12 and P90 (*n*_mice_=3). The data are presented as the mean±s.e.m. *P<0.05; **P<0.01; ***P<0.001 [one-way ANOVA test followed by Tukey's multiple comparisons test (B,C); two-tailed unpaired *t*-test (D)]. Scheme was prepared using the BioRender software.

Using western blot (WB) analyses, we first examined expression patterns of 5-HT1AR and 5-HT7R in the PFC, HP and RN at crucial developmental time points (i.e. P2, P12 and P90). As shown in Fig. 1, expression levels of 5-HT1AR in the PFC and HP at P2 and P12 were significantly higher compared to that in the RN, and this disparity persisted until P90 (Fig. 1A,B). For 5-HT7R, no significant differences in protein expression were observed between all structures at P2 and P12, whereas at P90 expression of this receptor in the RN was significantly lower than that in the PFC (Fig. 1A,C).

The gene expression profiles of both receptors were then analysed by quantitative RT-PCR (qRT-PCR). Using this approach, we were able not only to define mRNA expression profiles for 5-HT1AR and 5-HT7R in defined brain regions during development but also to calculate the relative expression ratio. These experiments revealed that the lowest expression levels of mRNA encoding both receptors in all three brain regions were at P12 (Fig. S1A). Interestingly, in the PFC and HP, 5-HT1AR mRNA expression reached the highest values at P90 (being significantly higher than those at P2), whereas expression of mRNA encoding 5-HT7R at P90 was a lot lower (Fig. S1B). The qRT-PCR analysis also revealed changes in expression ratios of 5-HT1AR to 5-HT7R (Fig. 1D). In the HP, a significant increase in 5-HT1AR levels over the 5-HT7R was observed at all developmental stages, reaching an ∼10-fold increase in 5-HT1AR expression over that for 5-HT7R at P90 (Fig. 1D). Also, in the PFC we obtained a similar distribution with a 5-to-1 ratio obtained for 5-HT1AR to 5-HT7R levels at P90. In contrast, in the RN at P2 we found ∼2-fold higher 5-HT7R than 5-HT1AR mRNA expression levels, whereas at P12 and P90 this difference was no longer visible (Fig. 1D). Taking into consideration our previously published model, which predicts relative concentration of monomers and dimers at any given defined expression ratio between 5-HT1AR and 5-HT7R (Renner et al., 2012), our results suggest that most of the 5-HT7R expressed in PFC and HP exists in form of heterodimers with 5-HT1AR. According to this experimentally validated model, the amount of 5-HT1AR–5-HT7R heterodimers at 10:1 ratio of 5-HT1AR to 5-HT7R will be ∼6-fold higher than the amount of 5-HT7R homodimers.

### Impact of chronic unpredictable stress on 5-HT1A and 5-HT7R expression levels

**Fig. 2.**
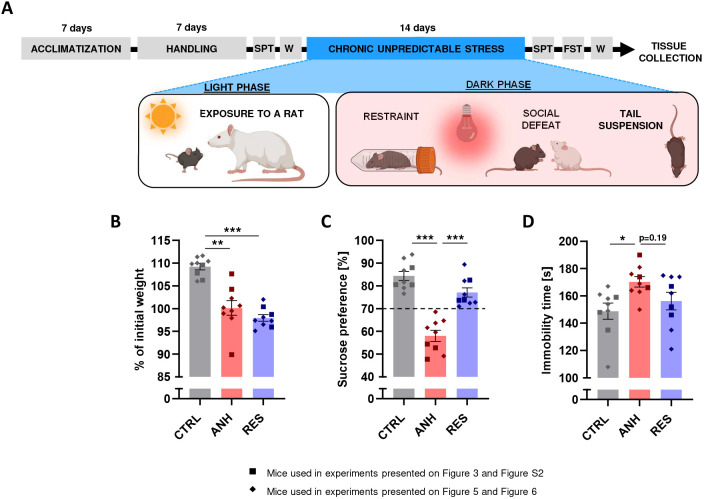
**Behavioural evaluation of depressive-like behaviour of ANH, RES and CTR animals following CUS.** (A) Schematic view of the experimental design of the CUS model (prepared using the BioRender software). SPT, sucrose preference test; FST, forced swimming test, W – body mass (weight) measurement. (B) Relative changes of initial body mass after CUS. (C) Results of SPT after CUS. (D) Results of FST after CUS in CTRL, ANH and RES animals. The data are presented as the mean±s.e.m. (*n*_mice_=9). *P<0.05; **P<0.01; ***P<0.001 [Brown–Forsythe ANOVA test followed by Tamhane's T2 multiple comparisons test (B); one-way ANOVA test followed by Tukey's multiple comparisons test (C,D)].

Because of the important role of both 5-HT1AR and 5-HT7R in the pathogenesis of depressive disorders (Albert and Vahid-Ansari, 2019; Bijata et al., 2022a), we next investigated their expression levels in the brain of mice subjected to a chronic unpredictable stress (CUS) protocol, which represents a suitable tool for modelling the depressive-like behaviour (Antoniuk et al., 2019; Bijata et al., 2022b). Following a 2-week stress protocol (Fig. 2A), two distinct populations of animals emerged – resilient (RES) and anhedonic (ANH) mice. Both populations exhibited reduced body mass gain compared to control mice (CTRL) (Fig. 2B). Moreover, ANH mice displayed a reduced sucrose preference in the sucrose preference test (SPT, Fig. 2C) and an increased immobility time in the forced swim test (FST, Fig. 2D) in comparison to CTRL animals. In contrast, RES mice did not differ from CTRL mice in these behavioural tests (Fig. 2C,D).

**Fig. 3.**
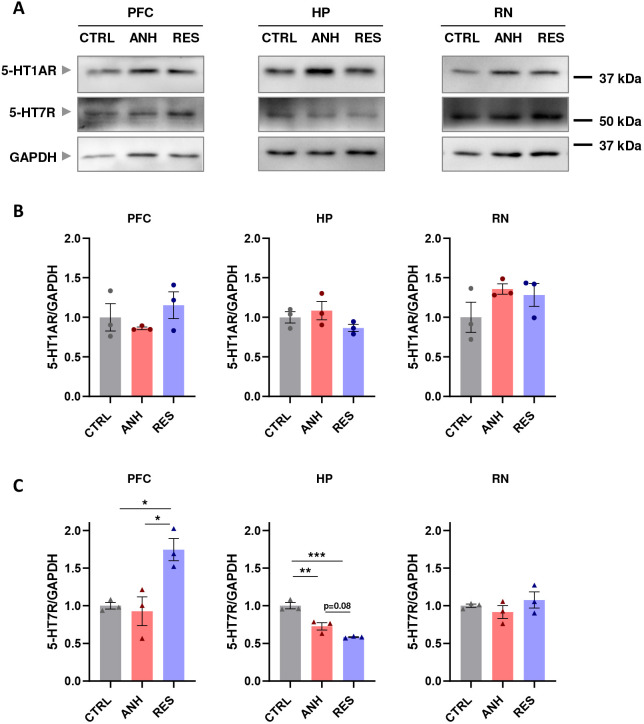
**Expression of 5-HT1AR and 5-HT7R in the PFC, HP and RN following CUS.** (A) Representative western blot showing 5-HT1AR, 5-HT7R and GAPDH expression in the PFC, HP and RN in CTRL, ANH and RES mice. Quantification of the 5-HT1AR (B) and 5-HT7R (C) expression in PFC, HP and RN of CTRL, ANH and RES mice (*n*_mice_=3). The data are presented as the mean±s.e.m. *P<0.05; **P<0.01; ***P<0.001 (one-way ANOVA test followed by Tukey's multiple comparisons test).

WB analysis revealed no significant differences in 5-HT1AR expression levels among CTR, ANH and RES mice in the PFC, HP and RN (Fig. 3A,B). However, expression of 5-HT7R differed significantly between the behavioural groups. In the PFC, we observed a significant increase in the amount of 5-HT7R in RES mice compared to that in both CTR and ANH animals (Fig. 3A,C). In the HP, expression of 5-HT7R in both ANH and RES mice was decreased in comparison to that in CTRL animals, whereas no changes in the 5-HT7R expression were observed in the RN (Fig. 3A,C). Interestingly, the qRT-PCR analysis revealed no differences between behavioural groups in the mRNA expression levels of 5-HT1AR and 5-HT7R across all examined brain structures (Fig. S2). These data demonstrate that the increased expression of 5-HT7R in the PFC accompanied with a decreased amount of the same receptor in the HP might be connected with development of the resilience phenotype.

### Impact of chronic unpredictable stress on heterodimerization of 5-HT1A and 5-HT7R

Next, we investigated whether the stress conditions can modulate hetero-oligomerization between 5-HT1AR and 5-HT7R in the brain. To this end, we performed an *in situ* proximity ligation assay (PLA), which allows detection of heterodimers for endogenously expressed receptors with cellular resolution.

**Fig. 4.**
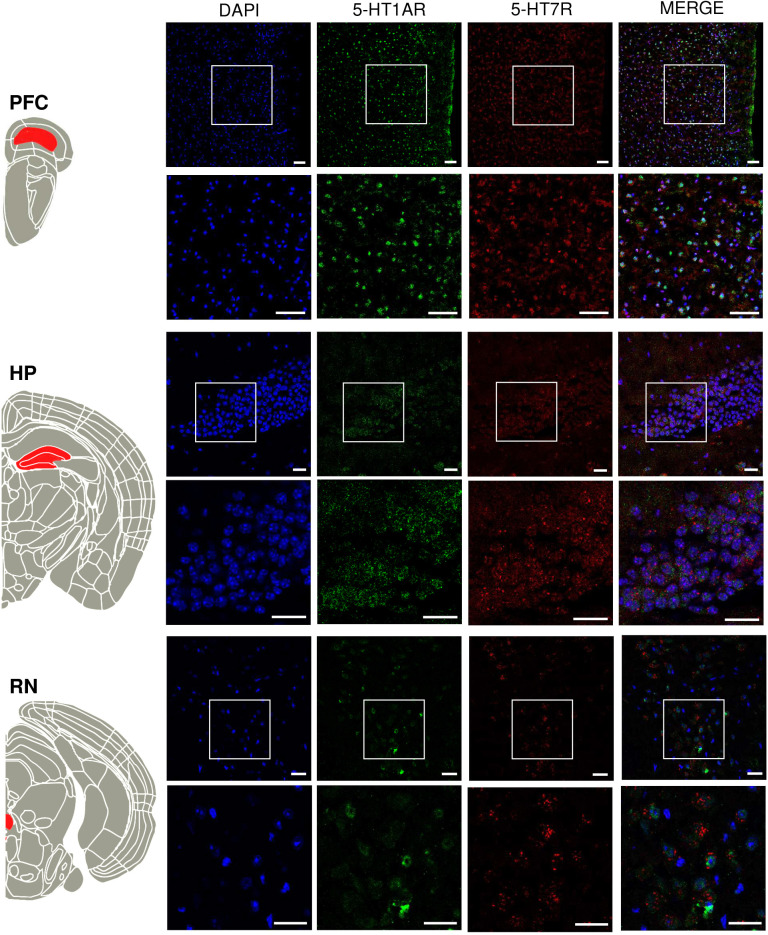
**Expression of 5-HT1AR and 5-HT7R in different brain areas.** Schematic presentation of brain regions analysed based on Allen Mouse Brain Common Coordinate Framework version 3 (Bakker et al., 2015) (on the left). PFC, prefrontal cortex; HP, hippocampus; RN, raphe nuclei. Representative immunohistochemical stainings from three repeats at P90 with DAPI (blue), 5-HT1AR (green) and 5-HT7R (red) are shown on the right. White squares mark regions enlarged at the bottom. Scale bars: 50 µm (PFC); 20 µm (HP and RN).

Given that the success of the PLA protocol implementation is strongly dependent on the specificity of immunohistological stainings, we first optimized staining protocols for the immunological detection of both receptors and confirmed colocalization of 5-HT1AR and 5-HT7R in all examined structures (PFC, HP and RN) (Fig. 4).

**Fig. 5.**
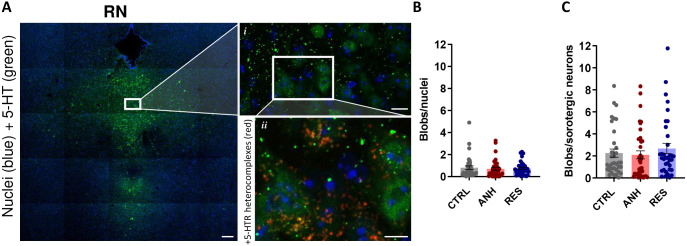
**Heterodimerization of 5-HT1AR and 5-HT7R in RN.** (A) An overview and an enlargement of immunohistochemical staining of 5-HT positive neurons (green) in the RN. DAPI is shown in blue. In addition, inset *ii* shows PLA signal (red). Scale bars: 100 µm (main image), 10 µm (inset *i*); 5 µm (inset *ii*). (B) Quantification of PLA-positive blobs per cell in RN of CTRL, ANH or RES animals. (C) Quantification of PLA-positive blobs restricted to 5-HT-positive cells in RN of CTRL, ANH or RES animals. The data are presented as the mean±s.e.m. (*n*_mice_≥5; CTRL=7, ANH=6, RES=5). Kruskal–Wallis test with Dunn's multiple comparison.

Subsequently, we applied an optimized PLA protocol (Fig. S3) to visualize and quantify formation of 5-HT1AR–5-HT7R complexes in the PFC, HP and RN of CTRL, ANH and RES mice. In the RN, we separately performed the PLA analysis in the general cell population and only on serotonergic neurons, which were identified by the immunostaining with antibody against serotonin. In the RN, no differences in heterodimerization (calculated as the number of PLA blobs per cell) were observed among CTRL, ANH and RES groups, both in the general population and in serotonergic neurons (Fig. 5). It is noteworthy that in serotonergic neurons of the RN, we observed at least twice as many 5-HT1AR–5-HT7R heterodimers than in the entire population of RN cells (Fig. 5B,C).

**Fig. 6.**
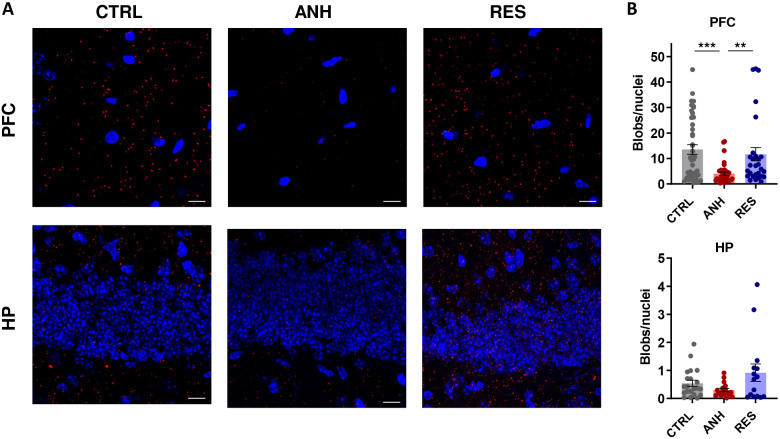
**Heterodimerization of 5-HT1AR and 5-HT7R in PFC and HP.** (A) Representative images of PLA results in the PFC (upper panel) and dentate gyrus of the HP (lower panel) of CTRL, ANH or RES animals. Scale bars: 10 μm. (B) Quantification of PLA-positive blobs per cell in both brain regions of CTRL, ANH or RES animals (*n*_mice_≥5; CTRL=7, ANH=6, RES=5). The data are presented as the mean±s.e.m. **P<0.01; ***P<0.001 (Kruskal–Wallis test with Dunn's multiple comparison).

In contrast to what was seen in the RN, the PLA analysis revealed significant differences in the levels of heterodimers in the PFC and HP. In the PFC of ANH mice, significantly fewer heterodimers were observed compared to in CTRL and RES mice (Fig. 6). In the HP, there were fewer heterodimers in ANH mice compared to the number in RES mice, with a statistically non-significant trend compared to CTRL mice (Fig. 6). Interestingly, when comparing only CTRL mice, we observed the highest number of heterodimers in the PFC (PFC 13.5±1.9; HP 0.5±0.1; RN 0.8±0.2, mean±s.e.m.).

## DISCUSSION

In the present study, we obtained higher protein expression levels for 5-HT1AR in the PFC and HP compared to in the RN in adult (i.e. P90) mice. Similar distribution patterns for 5-HT1AR have been mapped through receptor autoradiography utilizing [3H]-8-OH-DPAT and [3H]-WAY100635 in humans and mice brains, as well as by *in situ* hybridization in rats (Chalmers and Watson, 1991; Hall et al., 1997; Laporte et al., 1994; Wright et al., 1995). In the case of 5-HT7R, we observed lower receptor expression levels in the RN compared to in the PFC and HP.

Interestingly, the expression profile of both receptors dynamically changes during development, which is in line with previous studies (Duncan et al., 1999; Kobe et al., 2012; Renner et al., 2012). It has been suggested that variable expression of serotonin receptors and/or their downstream effectors might mediate the diverse effects of serotonin during development (Bonnin et al., 2006, 2007). It has also been shown that the coordinated changes in the expression of 5-HT7R and 5-HT1AR during neuronal maturation might be involved in the regulation of receptor functions (Prasad et al., 2019). Here, we found that there was an increase in 5-HT1AR expression across all examined structures at P90, whereas expression of 5-HT7R decreased in the HP and PFC starting from P12. This influences the mutual expression ratio of these receptors. In the HP and PFC, 5-HT1AR expression predominates over 5-HT7R reaching at P90 a 10-fold difference in the HP. Previous studies have demonstrated the involvement of the 5-HT7R in postnatal formation of synaptic connectivity in the PFC, where initially high expression of 5-HT7R later declines and is replaced by an increase in 5-HT1AR expression (Béïque et al., 2004a,b). These results are also in line with our previous studies showing a decrease in 5-HT7R expression in the HP during development (Kobe et al., 2012). The situation differs in the RN, where twice as many 5-HT7R as 5-HT1AR molecules were expressed at P2. During development, the difference in receptor expression gradually diminishes, resulting in similar expression levels starting from P12. The high expression of 5-HT7R in RN is an interesting observation, as previous studies have predominantly focused on 5-HT1A autoreceptors in this region, often neglecting the role of the 5-HT7R.

It is noteworthy that at both mRNA and protein levels, the amount of 5-HT1AR remained unchanged following exposure to CUS. This aligns with our previous findings where no alterations were observed in 5-HT1AR mRNA expression in Wistar rats after stress exposure (Gorinski et al., 2019). However, previous studies by others have presented contradictory finding. For example, results from Li et al. indicate an increase in 5-HT1AR expression in both the HP and PFC after 9 weeks of chronic stress, whereas Lin et al. show a reduction in 5-HT1AR protein levels in these brain regions following 4 weeks of chronic stress (Li et al., 2009; Lin et al., 2023). Interestingly, PET imaging based on [carbonyl-C-11]-WAY-100635 binding to 5-HT1AR has revealed higher binding potential in cortical areas, HP, amygdala and RN in males with major depressive disorder (MDD) compared to the control group (Kaufman et al., 2015). Conversely, some PET studies have shown decreased binding of [carbonyl-C-11]-WAY-100635 to 5-HT1AR in MDD-affected humans and in monkeys characterized by behavioural observations as depressed (Bhagwagar et al., 2004; Sargent et al., 2000; Shively et al., 2006). Furthermore, observations in individuals with depression and post mortem analyses of human brains indicate a disturbance in the balance of pre- and post-synaptic 5-HT1AR. Studies have demonstrated increased density of presynaptic 5-HT1AR, accompanied by a decrease in postsynaptic receptor population (Drevets et al., 2000; López-Figueroa et al., 2004; Sargent et al., 2000; Stockmeier et al., 1998). Observed discrepancies suggest potential complexities in the regulation of 5-HT1AR expression under chronic stress conditions. Possible factors contributing to such disparities could include variations in stress protocols in animal studies as well as methodological differences in the assessment of receptor expression.

**Fig. 7.**
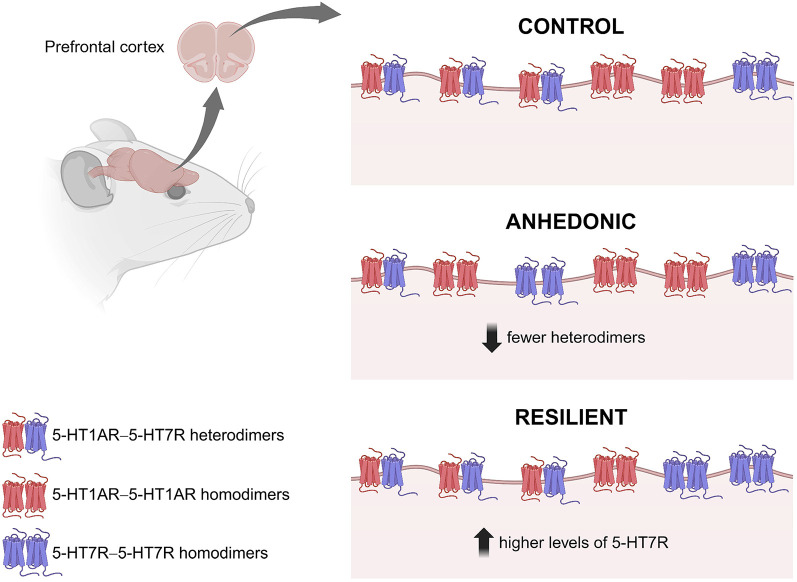
**Graphical summary of the present study.** Levels of 5-HT1AR–5-HT7R heterodimerization are reduced in the PFC of ANH animals, whereas in RES animals it remains at the same level as in control animals. This could be explained by increased expression of 5-HT7R in the PFC of RES mice. Scheme was prepared using the BioRender software.

In case of 5-HT7R, elevated levels of its mRNA have been reported in the HP but not in the cortical regions of rats exposed to CUS for 9 weeks (Li et al., 2009). By contrast, it has been shown that acute but not chronic immobilization stress increases 5-HT7R mRNA levels in the CA2 and CA3 regions of the rat HP (Yau et al., 2001), which is consistent with our findings. Of note, our study has demonstrated a considerable decrease in the expression 5-HT7R in the HP of RES mice. In our previous study, we showed that knockdown of the 5-HT7R via shRNA promotes resilience (Bijata et al., 2022a). The current findings support these observations, suggesting the resilience phenomenon can be connected with reduced expression levels of 5-HT7R in HP. Interestingly, we observed the opposite effect in the PFC (Fig. 7), where a significant increase in 5-HT7R protein levels occurred in RES animals.

Among other functions, such selective changes in 5-HT7R expression might be implicated in the heterodimerization of 5-HT1AR and 5-HT7R (Fig. 7). Heterodimerization provides an additional control for signal transduction as well as for the fine-tuning of cellular processes. The formation of heterodimers might also alter the pharmacological properties of the receptors, influencing their affinity and selectivity for ligands. Heterodimerization depends on various factors, such as receptor expression levels, and might be modulated upon pathological conditions (Quitterer and AbdAlla, 2019; Schellekens et al., 2013). Stress is known to trigger intricate changes in the CNS, affecting neurotransmitter systems and receptor activities. We and others have shown that stress can influence the functioning of both 5-HT1AR and 5-HT7R (Bijata et al., 2022a; Gorinski et al., 2019; Yohn et al., 2017). Therefore, understanding the alterations in the heterodimerization of these receptors under stress conditions is crucial for unravelling the mechanisms behind stress-related psychiatric disorders and might pave the way for targeted therapeutic interventions.

According to our results, the PFC is the region exhibiting the highest abundance of 5-HT1AR–5-HT7R heterodimers. Of note, the level of heterodimerization was significantly reduced in ANH animals, whereas in RES animals, 5-HT1AR–5-HT7R heterodimerization remains at the same level as in control animals (Fig. 7). This might be due to the elevated expression levels of 5-HT7R in RES mice. According to our experimentally validated model predicting relative concentration of dimers at any given defined expression ratio between 5-HT1AR and 5-HT7R (Renner et al., 2012), a selective increase in 5-HT7R expression obtained in PFC of RES animals will result in higher number of heterodimers, which might be implicated in the development of resiliency phenomenon. Multiple ongoing studies (including our own) are attempting to address the factors determining resilience or susceptibility to stress in detail, in particular because of their enormous clinical impact. Despite a uniform genetic background and consistent stressors, differences in stress responses, finally leading to anhedonia or resiliency can arise from individual variations in epigenetics, early life experiences, homeostatic mechanisms and adaptive coping strategies as well as post-translational modifications (Bączyńska et al., 2023 preprint; Cathomas et al., 2019; Highland et al., 2019; Peña et al., 2019; Stainton et al., 2019; Wu et al., 2013).

What could be a possible mechanism, by which 5-HT1AR–5-HT7R hetero-oligomerization levels is selectively reduced in PFC of only ANH animals even the expression levels of both receptors are not affected in comparison with a control? One possible reason could be differences in palmitoylation. Indeed, both 5-HT1AR (Papoucheva et al., 2004) and 5-HT7R (Kvachnina et al., 2009) undergo palmitoylation. For 5-HT7R, palmitoylation is involved in the regulation of its agonist-independent Gαs-mediated constitutive activity. In case of the 5-HT1AR, palmitoylation is necessary for Gαi coupling and effector signalling. We also shown that palmitoylation of 5-HT1AR does not directly modulate oligomerization of 5-HT1AR, but serves as a targeting signal responsible for the retention of the receptor within membrane microdomains (Kobe et al., 2008; Renner et al., 2007; Woehler et al., 2009). More importantly, palmitoylation of the 5-HT1AR in the PFC is attenuated in rodent models of depression as well in post-mortem brain samples from individuals with MDD who have died by suicide (Gorinski et al., 2019). These data, together with results obtained in the present study, suggest that reduced 5-HT1AR–5-HT7R heterodimerization might be explained by decreased 5-HT1AR palmitoylation in PFC of ANH animals. This could result in an artificial relocation of 5-HT1AR outside of the membrane subdomain, resulting in a decreased probability of its interaction with 5-HT7R (Renner et al., 2007). It would be therefore interesting to compare palmitoylation levels of both receptors in ANH and RES animals in the future studies.

## MATERIALS AND METHODS

### Animals

Ten-week-old male C57BL/6J mice (Medical University of Bialystok, Poland) were individually housed under a reverse 12 h light–12 h dark cycle (lights on at 8:00 PM) with food and water available *ad libitum*. Male 12-week-old CD1 mice (Janvier Labs, France) were used as resident intruders in the social defeat stress procedure and kept with the stressed C57BL6J mice in the same animal room. Male 12-week-old Wistar rats (Mossakowski Medical Research Institute, Polish Academy of Sciences, Warsaw, Poland) were used for predator stress. Male naïve P2, P12 and P90 C57BL/6J mice (Hannover Medical School, Germany) were used for isolation of HP, prefrontal cortex and raphe nuclei. All animal procedures were performed according to the guidelines of the Polish Ethical Committee on Animal Research (permission no. 132/2016).

### CUS

To evaluate depressive-like behaviour in the mouse model, we used the chronic unpredictable stress paradigm (CUS) and behavioural evaluation as described previously (Bączyńska et al., 2023 preprint; Bijata et al., 2022a). We have described a highly reproducible protocol with all the relevant technical details previously (Bijata et al., 2022b). Briefly, before CUS, C57BL/6J mice were subjected to 2 weeks of room acclimatization, consisting of 1 week of handling. Mice were weighed and their baseline sucrose preference was measured before the CUS procedure. Then, based on their baseline parameters, mice were assigned to a control and stress group housed in two separate rooms. The 2-week CUS protocol consisted of two out of three different types of stressors chosen in a semirandom manner and applied daily during the dark phase under red light in the following sequence of restraint stress, tail suspension and social defeat stress, with an intersession of at least 3 h. During each light phase during the stress protocol, the mice were exposed to a rat. To stabilize glucocorticoid levels after the last exposure to a stressor, the mice were left undisturbed overnight before beginning the sucrose preference test. Thus, 16 h after the last stressor, the mice underwent a sucrose preference test, resulting in the determination of sucrose preference 24 h after the last stressor, and thereafter, the body mass measurements and the forced swim test were performed. All mice were killed at 12–16 h after the behavioural evaluation (36–38 h after the last stressor). The experimental design is outlined in Fig. 2A.

#### Restraint stress

The mice were placed inside a plastic tube (26 mm internal diameter) for 2 h during the dark phase.

#### Tail suspension stress

The mice were subjected to the tail suspension procedure by being hanged from the tails with adhesive tape for 40 min during the dark phase. To prevent the mice from climbing their tails, plastic cylinders (4 cm×0.5 cm) were placed at the base of their tails.

#### Social defeat stress

During each 30 min social defeat session performed in the dark phase, aggressive CD1 mice were placed in the home cages of C57BL/6J mice in the stress group. CD1 aggressive mice were selected as the CD1 mice that attacked C57BL/6J mice in less than 60 s without injuring them. During each session, the C57BL/6J mice exhibited signs of social defeat stress, such as a flight response, submissive posture and audible vocalization. If the mice in the stress group did not display signs of social defeat stress, then the CD1 mouse was changed to another CD1 mouse. In rare cases of physical harm that occurred between pairs of mice, aggressive CD1 individuals were immediately removed from the cage of the C57BL/6J-resident mice.

#### Predator stress

The mice were individually introduced into transparent well-ventilated cylinders (15 cm×8 cm) with food and bedding. The cylinders were then placed for 12 h (08:00 PM – 08:00 AM) into a rat home cage that contained a rat during the light phase. For the rest of the day (08:00 AM – 08:00 PM), the mice and rats were housed in their home cages in the same experimental room.

### Behavioural tests

#### Sucrose preference test

Mice were given free-choice access to 1% sucrose solution and water that were provided in identical bottles for 8 h during the dark phase under a reverse light–dark cycle. The percentage of sucrose preference was calculated as follows: sucrose preference=[mass sucrose solution/(mass sucrose solution+mass water)]×100%. The consumption of water and sucrose solution was estimated simultaneously in the control and experimental groups by weighing the bottles. To eliminate possible bias from side preference, the positions of the bottles were changed after 4 h of the test. At 24 h before the baseline SPT performed before the CUS procedure (SPT0) was undertaken, 2.5% sucrose solution was given to all animals for 2 h to prevent the possible effects of taste neophobia. The other conditions of the test were performed as previously described (Bijata et al., 2022b). A sucrose preference <70% in mice in the stress group measured after the 8 h SPT (24 h after cessation of the stress procedure) was the criterion for ‘anhedonia’ defined by difference between control and stressed group >2xSD. ANH mice were previously shown to display depressive-like behaviour (Bijata et al., 2022b; Strekalova and Steinbusch, 2010; Strekalova et al., 2004). None of the control animals exhibited <70% sucrose preference in SPT. Stressed mice with sucrose preference >70% at the end of the CUS experiment were defined as resilient animals.

#### Forced swim test

Cylindrical glass containers (20 cm×40 cm) were filled with warm water (∼27°C) to a depth of 15 cm. The test was conducted under red light during the dark phase of the light–dark cycle 1 h after SPT following CUS. Animals were acclimatized for 1 h to the room where the FST was performed. Each mouse was placed in the water for 6 min for a single swim session. The total amount of time spent floating during the last 4 min was manually assessed.

### qRT-PCR

The mRNA was purified using the RNeasy^®^ Plus Mini Kit (cat. no. 74136, Qiagen). Briefly, brain tissue from different regions (PFC, HP and RN) was lysed in the RNeasy lysis buffer (RLT) containing DTT. Further steps were performed according to the manufacturer's instructions. mRNA concentration was measured using a NanoDrop 2000. Isolated mRNA was transcribed into cDNA using the SuperScript III First-Strand Synthesis System (reverse transcription; cat. no. 18080-051, Invitrogen) according to the manufacturer's protocol. Expression levels of 5-HT1AR and 5-HT7R were analysed on a StepOne System (Applied Biosystems) using TaqMan Universal PCR Master Mix (cat. no. 4324018, Applied Biosystems) and the Gene Expression Assays [Thermo Fisher Scientific; Mm01204045_m1 (for 5-HT7R) and Mm00434106_s1 (for 5-Ht1AR)]. For quantitative analysis, GAPDH expression levels were analysed in parallel with the following primer/probe sequences: GAPDH, fw 5'-TGCACCACCAACTGCTTAGC-3′; rev 5′ GGCATGGACTGTGGTCATGAG-3′; probe 5′ /6FAM/CCCTGGCCAAGGTCATCCATGACAAC/TAMRA/-3′ (Sigma). Calculation of relative mRNA levels was performed by using the ΔΔCt method.

### Western blotting

Brain tissue from the PFC, HP and RN was homogenized in homogenization buffer (10 mM HEPES pH 7.4, 5 mM EGTA, 1 mM EDTA and 0.32 M sucrose). The membrane fraction and soluble fraction were separated by applying the following centrifugation steps: 300 ***g*** (5 min at 4°C), 800 ***g*** (5 min at 4°C) and 20,000 ***g*** (1 h at 4°C). The obtained pellet was lysed using lysis buffer (150 mM NaCl, 50 mM Tris-HCl pH 7.4, 5 mM EDTA, 0.1% SDS and 0.2% Triton X-100). Protein concentration was measured with a Pierce™ BCA protein assay kit. Equal amounts of proteins were subjected to dodecyl-SDS-PAGE using the Biometra SDS-PAGE system. SDS-PAGE-separated proteins were transferred using the semi-dry western blot system using nitrocellulose membranes. Membranes were blocked in the 5% non-fatty dry milk in TBS with 0.05% (v/v) Tween 20 (TBST) for 1 h at room temperature with agitation. Afterward, membranes were incubated with primary antibodies (5-HT1A, Alomone ASR-021 1:250 in 3% BSA in TBST; 5-HT7R, Abcam AB128892 1:1000 in 3% BSA in TBST; GAPDH, Millipore MAB374 1:10,000 in the 5% non-fatty dry milk in TBST) at 4°C overnight. The blots were washed three times with TBST and then incubated for 1 h with a peroxidase-conjugated secondary antibody diluted 1:5000 in TBST containing 5% non-fatty dry milk (goat anti-rabbit IgG, Thermo Fisher Scientific, cat. no. 31460; and rabbit anti-mouse IgG, Thermo Fisher Scientific, cat. no. 31455). After washing, membranes were developed with SuperSignal Femto chemo-luminescence substrate and imaged with the FUSION XL Imager (Peqlab). See also Fig. S4 for original uncropped western blots used to create Figs 1 and 3.

### Immunohistochemistry

C57BL/6J mice were anesthetized and perfused intracardially with 4% (w/v) paraformaldehyde. Brains were removed, post-fixed in 4% paraformaldehyde at 4°C for 4 h, cryoprotected in 30% (w/v) sucrose solution. Brain coronal sections (30 μm) were cut on a cryostat and processed for free-floating histochemistry. The sections were pre-incubated in the sodium citrate buffer (80°C; 30 min) washed twice in PBS (room temperature, 2×5 min) and with methanol (−20°C; 5 min). Brain slices were incubated in a blocking buffer containing 0.1% (w/v) Triton X-100 and 5% (w/v) donkey serum. After 30 min incubation at room temperature, the sections were incubated with the primary antibodies [1:100 mouse monoclonal anti-5-HT1A receptor (cat. no. MAB11041, Millipore); 1:100 rabbit monoclonal anti-5-HT7 receptor (cat. no. 24430, Immunostar); 1:1000 goat monoclonal anti-serotonin (cat. no. ab66047, Abcam)] for 48 h at +4°C. Control experiments employed only secondary antibodies. Primary antibodies were extensively washed with PBS (room temperature, 3×10 min), and stained with the indicated fluorescent-labelled secondary antibodies. The secondary antibodies were applied for 1 h at room temperature [Alexa Fluor 488-conjugated donkey anti-mouse-IgG (1:400; cat. no. 715-545-150, Jackson ImmunoResearch); Alexa Fluor 594-conjugated donkey anti-rabbit-IgG (1:400; cat. no. 711-585-152, Jackson ImmunoResearch); Alexa Fluor 488-conjugated donkey anti-goat-IgG (1:2000; cat. no. A-11055, Invitrogen)]. The sections were mounted in Fluoroshield mounting medium with DAPI (cat. no. ab104139, Abcam).

### *In situ* PLA

To study the 5-HT1AR−5-HT7R interaction, an *in situ* proximity ligation assay (PLA) was performed according to the previously protocol (Borroto-Escuela et al., 2015) using the Duolink insitu PLA Kit (DUO92002, DUO92004, DUO92008; Sigma) with minor modifications. Briefly, brain sections were incubated with the primary antibodies as described in the Immunohistochemistry section. Afterward, the slices were washed three times with PBS and incubated with secondary antibodies conjugated to Plus and Minus PLA oligonucleotide arms for 2 h at 37°C in a humidity chamber. In case of interaction, DNA probes become located in a close proximity, which allows for rolling circle DNA amplification. The unbound proximity probes were removed by washing the slides three times with PBS at room temperature under gentle agitation. Next, sections were incubated with the hybridization−ligation solution (0.025 U/μl) and the amplification mixture containing fluorophore probes (λ_ex_=594 nm and λ_em_=624 nm; 125 units/μl) and incubated in a humidity chamber at 37°C for 60 and 100 min, respectively. Excess ligation solution was removed by applying the washing buffer A. In the last step, the sections were washed twice in the dark, for 10 min each, with the washing buffer B at room temperature. The free-floating sections were mounted on a microscope slide in mounting medium containing DAPI (VectaShield, Sigma). The sections were protected from light and stored for several days at −20°C before confocal microscopy analysis.

### Microscopy and images analysis

All data were collected on a Zeiss LSM 780 microscope controlled with ZEN 2012 software. The imaging was performed in the lambda mode resulting in emission spectra collection. This approach enables the unmixing of the different fluorophore spectra directly during the measuring. Images were acquired in online fingerprinting mode. DAPI, Alexa Fluor 488, Alexa Fluor 594, Alexa Fluor 647, Texas Red and autofluorescence reference spectra were used. *Z*-stacks were collected in order to get even information about the signal distribution, and maximum projection was applied for analysis. Immunohistochemical stainings were collected applying the following configurations: (1) 40×/1.2 NA water immersion objective (bit depth 16-bit, zoom 2.0, 30.00 μm); (2) 20×/0.8 NA air immersion objective (bit depth 16-bit, zoom 0.7, 20.00 μm). Images processing was performed using Fiji software.

The number of cell bodies and serotonergic cells was calculated using the Cell Counter plugin upon manual marking. The number of 5-HT1AR–5-HT7R hetero-complex blobs, which were detected based on their fluorescence spectra, was automatically evaluated by applying the function ‘Analyze particles’. Image processing included the following steps: (1) background setting; (2) threshold adjustment; (3) defining particles size; (4) assessment of the number of 5-HT1AR-5-HT7R hetero-complex blobs. The final calculation was performed according to the formulas: blobs/cell, 5-HT1AR–5-HT7R hetero-complex blobs number/cell body number, and blobs/serotonergic neuron, 5-HT1AR–5-HT7R hetero-complex blobs number/serotonergic neurons cell number.

### Statistical analysis

Statistical differences were calculated using GraphPad Prism8 software. Details of the statistical analysis are specified in the figure legends. In order to analyse statistical differences a two-tailed unpaired *t*-test or a one-way ANOVA with the Tukey post-hoc test were applied if not indicated differently. The western blots were quantitatively analysed by the sum of replicates (each data point on a replicate is divided by the sum of the values of all data points in that replicate) (Degasperi et al., 2014). Protein and mRNA expression signals were normalized to the loading control and housekeeping gene, respectively. For relative comparisons, the control mean was set equal to one. The mean+s.e.m. per experiment was used for statistical analyses. Statistical significance is annotated as *P<0.05, **P<0.01, ***P<0.001, ****P<0.0001. Data were considered significantly different when *P*<0.05. Western blot analyses were not conducted by a researcher who was unaware of the experimental conditions manner due to the necessity of arranging the samples on the gel in a specific order.

## Supplementary Material



10.1242/joces.262219_sup1 Supplementary information
