# Supplementary material for: The interplay of serotonin 5-HT1A and 5-HT7 receptors in chronic stress
Source: J Cell Sci. 2024 Oct 11;137(19):jcs262219. doi: 10.1242/jcs.262219 (PMC11491811; doi:10.1242/jcs.262219)
Supplement: Supplementary information [file joces-137-262219-s1.pdf]

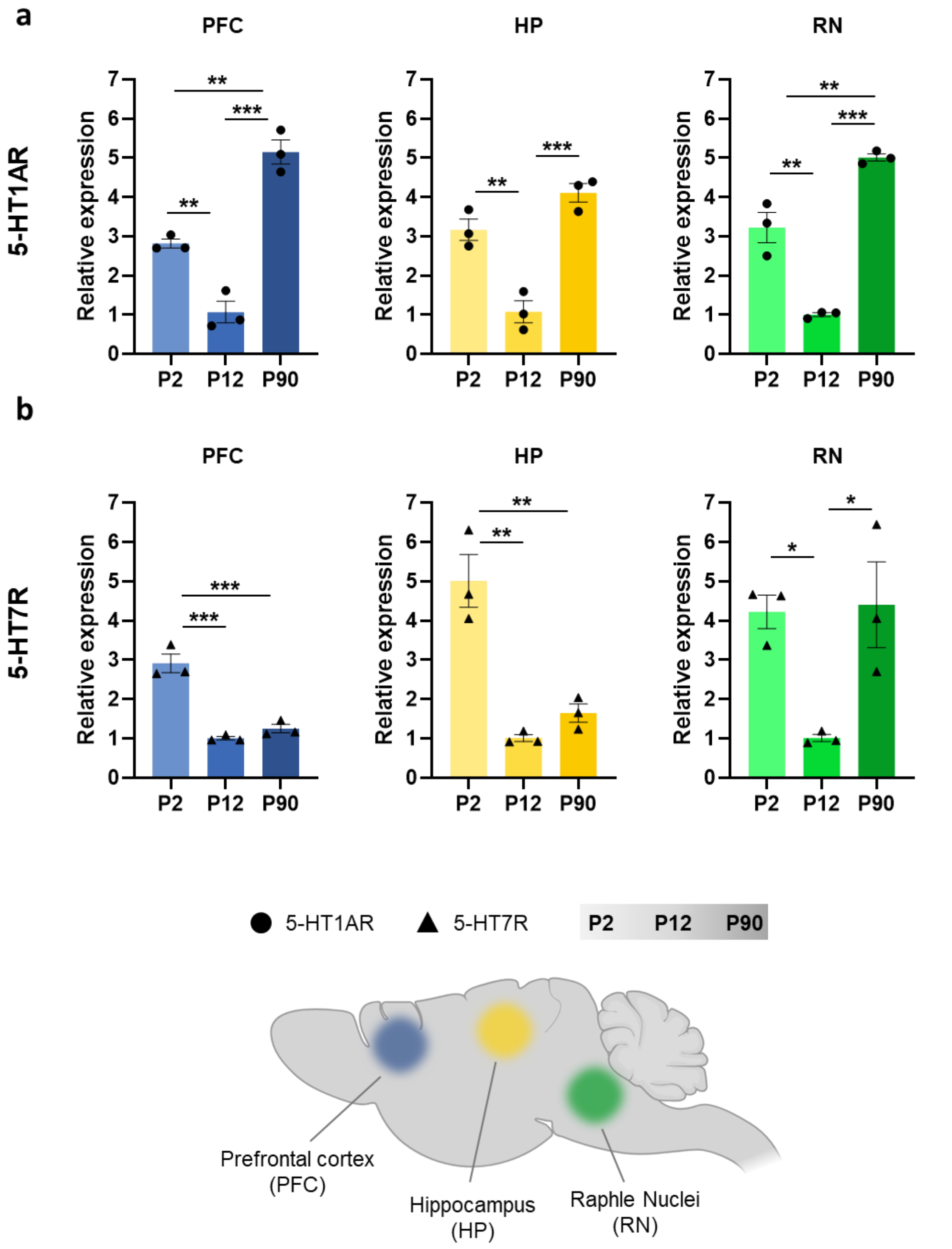

Fig. S1. Expression of mRNA encoding 5-HT1AR and 5-HT7R in the prefrontal cortex (PFC), hippocampus (HP), and raphe nuclei (RN) during development. Expression of mRNA encoding 5-HT1AR **(A)** and 5-HT7R **(B)** in indicated brain regions at postnatal day 2 (P2), 12 (P12) and 90 (P90); $n_{mice} = 3$. The data are presented as the mean ± SEM. $*p < 0.05$; $**p < 0.01$; $***p < 0.001$. (One-way ANOVA test followed by Tukey's multiple comparisons test). Scheme was prepared using the BioRender software.

Journal of Cell Science • Supplementary information

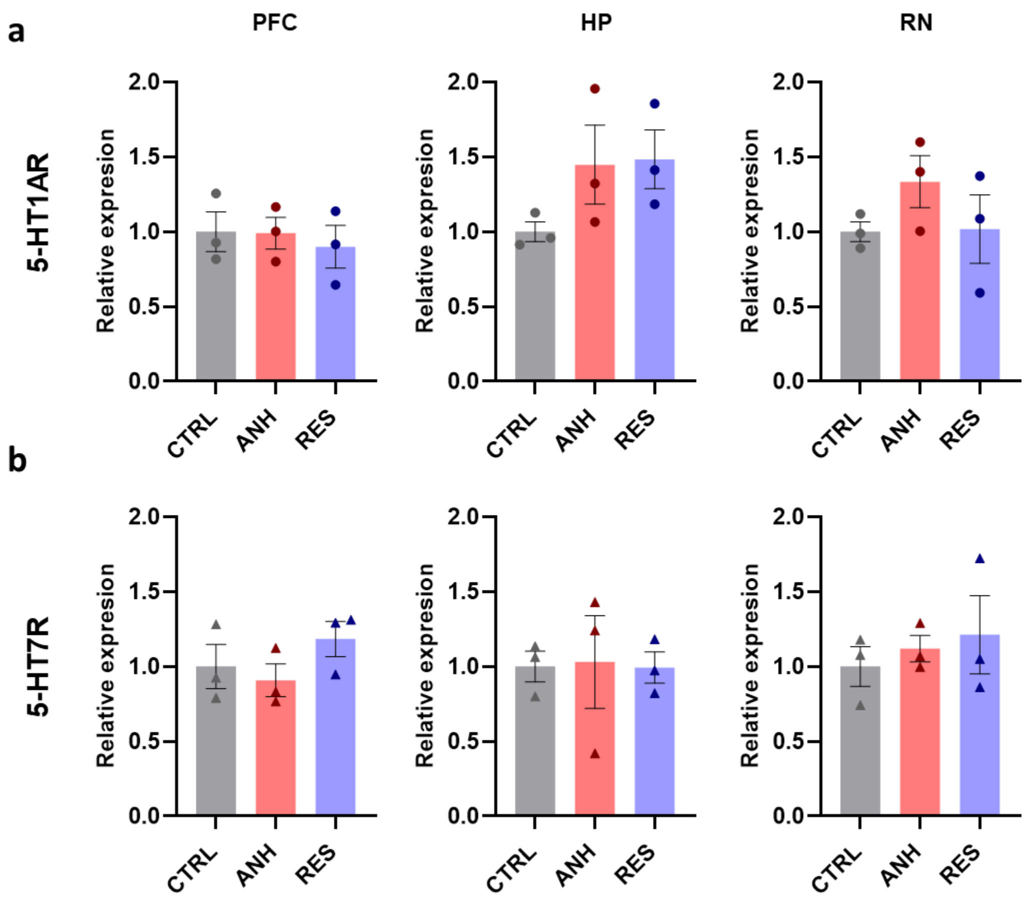

Fig. S2. Expression of mRNA encoding 5-HT1AR and 5-HT7R in the prefrontal cortex (PFC), hippocampus (HP), and raphe nuclei (RN) following chronic unpredictable stress (CUS).

Expression of genes encoding 5-HT1AR **(A)** and 5-HT7R **(B)** in HP, PFC and RN in control (CTRL), anhedonic (ANH) and resilient (RES) mice. The data are presented as the mean ± SEM. (One-way ANOVA test followed by Tukey's multiple comparisons test).

Journal of Cell Science • Supplementary information

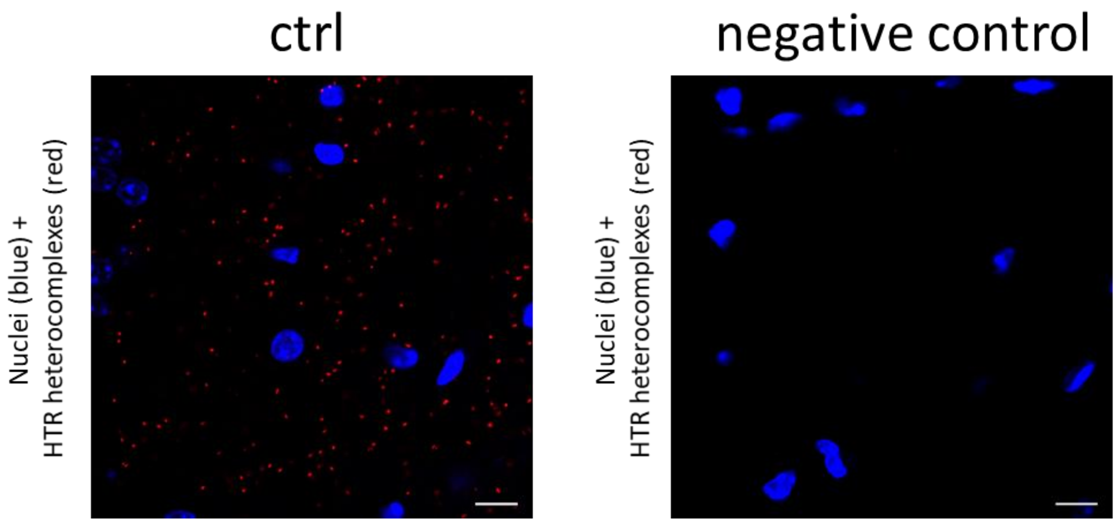

Fig. S3. Verification of the optimized PLA protocol.

PLA signal (red) and DAPI (blue) in raphe nuclei. In control (CTRL) experiments, PLA protocol was carried out as described in “Materials and methods” section, while in negative control PLA procedure was conducted in the absence of primary antibody. Both experiments were performed using brain slicers from CUS control animals at P120. Scale bar: 10µm.

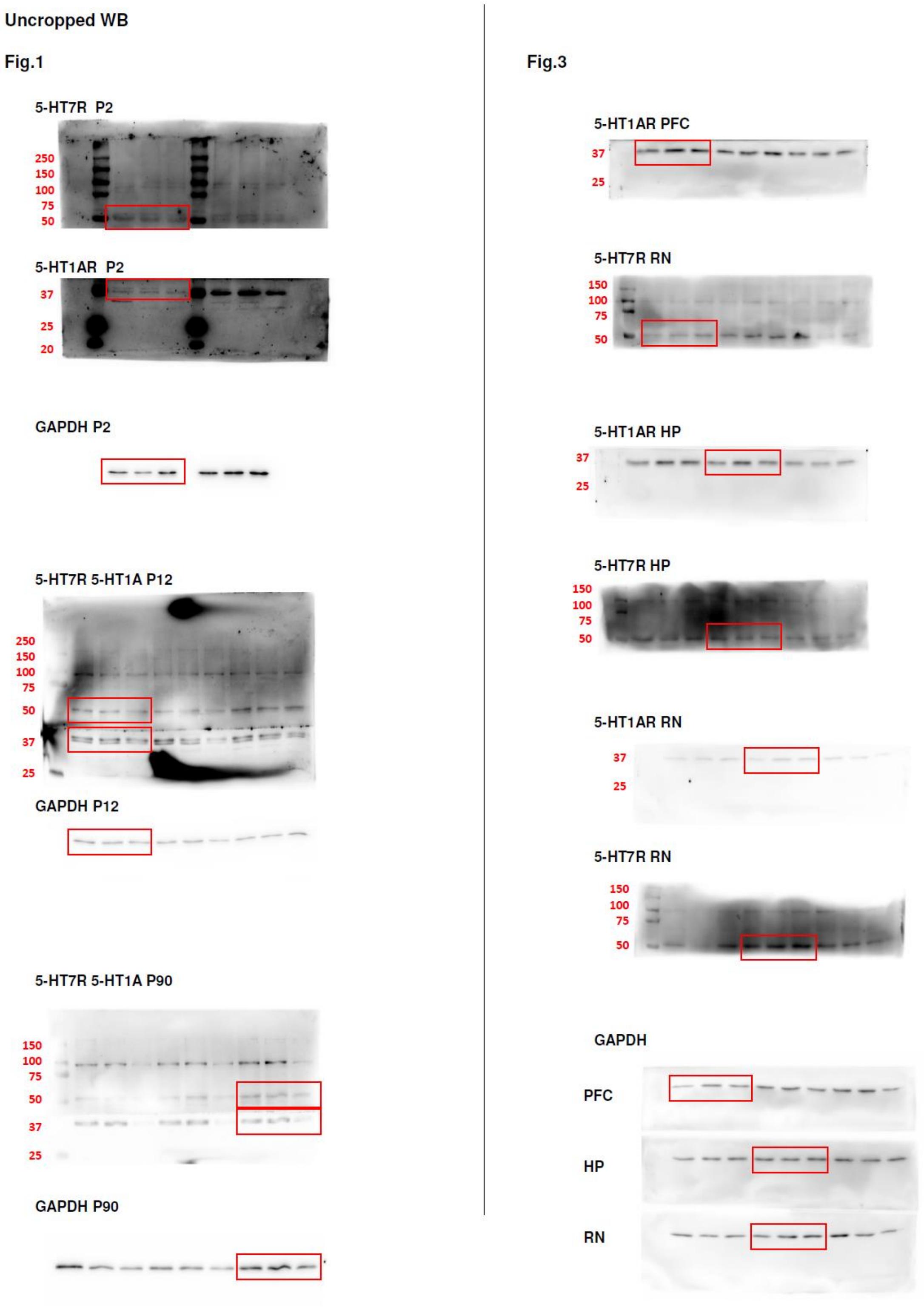

Fig. S4. Blot Transparency.

Uncropped Western blot used for creation of images shown in Figs. 1 and 3.

Journal of Cell Science • Supplementary information
