# Supplementary material for: The interplay of serotonin 5-HT1A and 5-HT7 receptors in chronic stress
Source: J Cell Sci. 2024 Oct 11;137(19):jcs262219. doi: 10.1242/jcs.262219 (PMC11491811; doi:10.1242/jcs.262219)
Supplement: Supplementary file 1 [file joces-137-262219_review_history.pdf]

The interplay of serotonin 5-HT1A and 5-HT7 receptors in chronic stress

Monika Bijata, Alexander Wirth, Jakub Wlodarczyk and Evgeni Ponimaskin
DOI: 10.1242/jcs.262219

Editor: Giampietro Schiavo

Review timeline

Original submission:	25 April 2024
Editorial decision:	28 June 2024
First revision received:	5 August 2024
Editorial decision:	3 September 2024
Second revision received:	4 September 2024
Accepted:	11 September 2024

Original submission

First decision letter

MS ID#: JOCES/2024/262219

MS TITLE: The interplay of serotonin 5-HT1A and 5-HT7 receptors in chronic stress

AUTHORS: Monika Bijata, Alexander Wirth, Jakub Wlodarczyk, and Evgeni Ponimaskin

We have now reached a decision on the above manuscript.

To see the reviewers' reports and a copy of this decision letter, please go to: https://submit-jcs.biologists.org and click on the 'Manuscripts with Decisions' queue in the Author Area. (Corresponding author only has access to reviews.)

As you will see, the reviewers raise a number of substantial criticisms that prevent me from accepting the paper at this stage. They suggest, however, that a revised version might prove acceptable, if you can address their concerns. If you think that you can deal satisfactorily with the criticisms on revision, I would be pleased to see a revised manuscript. We would then return it to the reviewers.

Please ensure that you clearly highlight all changes made in the revised manuscript. Please avoid using 'Tracked changes' in Word files as these are lost in PDF conversion.

I should be grateful if you would also provide a point-by-point response detailing how you have dealt with the points raised by the reviewers in the 'Response to Reviewers' box. Please attend to all of the reviewers' comments. If you do not agree with any of their criticisms or suggestions please explain clearly why this is so.

Reviewer 1

Advance summary and potential significance to field

© 2024. Published by The Company of Biologists under the terms of the Creative Commons Attribution License (https://creativecommons.org/licenses/by/4.0/).

This paper describes regional brain changes in 5UT 1 and 7 receptors in response to Chronic Unpredictable Stress. This paradigm yields populations of both susceptible and resistant animals.

Chronic unpredictable stress increases 5HT7R in the PFC of animals resistant to that stress, and this is one of the most robust findings in this study. Resilient animals also show an increase in heterodimerization in all three brain regions examined in this study.

Significant decreases in 5HT1/5HT7 dimerization occurred in 2 of the three regions studied, but CUS causes this independent of effects on “mood” in the rodents.

The experiments performed use sophisticatd methodology and multiple methods are used for detection.
Certainly this increases validity of the data.

The issue for this reviewer is “So, what does this mean”. Frankly the study would be more complete with an examination of antidepressant treatment after CUS. Would it affect both resistant and susceptible animals.
While this would make the study more interesting, it would be unfair to ask the authors to add these studies as they likely would require as much time is the current study. Furthermore, while this study raises several unanswered questions, it is complete in it’s current form.

Note grammatic errors, including p4 “Our study demonstrated that even though the expression levels ….

P9 ‘The situation differs in the RN, where twice as many 5-HT7R as 5-HT1AR were …

Comments for the author

see above

Reviewer 2

Advance summary and potential significance to field

General comments:
The authors took a challenge to study the dynamics of 5-HT1AR and 5-HT7R expression and their heterodimerization upon chronic stress. In parallel, they studied receptors expression pattern in mouse brain during development. Authors found no differences in ratio between 5-HT1AR and 5-HT7R expression in young mice, but increased levels of 5HT1AR to 5-HT7R in 3-month-old mice. In a CUS model, they observed no differences in 5HT1AR expression, increased expression 5HT7R expression in PFC of resilient animals.
Furthermore, they found a decreased number of heteromeric 5HT1AR/5HT7R complexes in brains of anhedonic mice in contrast to resilient mice after CUS.
Despite the high relevance of the topic, the tested hypothesis is not clear formulated, and the goal of the study is not defined.

Comments for the author

General comments:
Despite the high relevance of the topic, the tested hypothesis is not clear formulated, and the goal of the study is not defined. If the hypothesis is that: “The expression of the 5HT1AR is increased with aging and associated with depression, which can be attenuated by heteromerization with 5HT7R observed in resilient mice”, then it must be stated very clear both, in abstract and in the introduction, and confirmed experimentally. Now, two stories to be observed: one is about receptors expression pattern during development and depression, another – about receptors heteromerization in depression. If the main hypothesis is that receptors heteromerization during development is an explanation for the resilience against stress in adult mice, then it should be clearly identified and confirmed experimentally.

© 2024. Published by The Company of Biologists under the terms of the Creative Commons Attribution License (https://creativecommons.org/licenses/by/4.0/).

An explanation should be provided in the section CUS model, why some offspring could become anhedonic and others resilient.
A graphical abstract would be very helpful to follow the process of proving of hypotheses with provided experimental design.

Minor Comments:
1. Abstract
Page 2 Paragraph 2 Line 1 and 3: “mice” instead “animals” is clearer for the reader and simplify the search in data bases for mice studies. The authors should specify the animal species here.
Page 2 Paragraph 2 Line 2: typo, should be „stages“ instead of „staged“

2. Introduction
Page 4 Paragraph 2 Line 7-10: Here, the authors described only results in a CUS model, as a main finding.
However, in the abstract they also described another finding - an increased expression of the 5HT1AR in three-months-old mice. This finding must be integrated also here with an interpretation/explanation of the results in a short form.

3. Results
In general, the same order of the following list by defining analyzed brain regions through the manuscript:
“prefrontal cortex, hippocampus and raphe nuclei” will be very helpful for understanding of the tested hypothesis, results and their interpretation.
Figure 1 Panel a: The abbreviation “HP” should be the same as in the text: “HIP”, also in the figure legend and through the whole manuscript. The number of WB experiments should be provided. In general, for all figures:
letters format should be the same on the panels and figure legend: capital or not. This will simplify the reading.
Page 5 Paragraph 2 Line 8 (expression ratios 5HT1AR and 5HT7R): The Figure 1D should be referred already in this sentence for a better understanding of the analysis.

Figure 1 Figure legend Panel (D): Typo, “ö” and “5-H7RR”

Figure S1. The number of animals used for the analysis should be provided. ”HP” should be “HIP” as in the text.

Page 5 Paragraph 2: The authors supposed: “Taking into consideration our previous observation that the 5-HT7R possesses the highest affinity to form homodimers, followed by the 5-HT7R/5-HT1AR heterodimers and 5-HT1AR/5-HT1AR homodimers [17], our results suggest that the most of 5-HT7R expressed in PFC and HIP exist in form of heterodimers with the 5-HT1AR.” This is contradictory, if the 5-HT7R possesses the highest affinity to form homodimers, why the most of 5-HT7R expressed in PFC and HIP should exist in form of heterodimers with the 5-HT1AR and not in homodimers? The authors must provide more convenient explanation.

Page 5 Paragraph “Impact of Chronic Unpredictable Stress on 5-HT1A and 5-HT7R Expression Levels”: if there no special reasons for capital letters in “Expression Levels” then it should be in the same format as in the whole manuscript.

Figure 2. The number of animals per each group should be provided. If the Figure 2a has been created by using some software such as BioRender, it should be mentioned in the figure legend. The abbreviations “SPT”, “W”, “FST” should be provided in the figure legend.
Figure 3 Panel a: The abbreviation “HP” should be the same as in the text: “HIP”, also in the figure legend. The number of WB experiments should be provided.
The quality of the WB of 5HT7R in PFC, HP and RN is very low and should be improved. Also, the expression of the 5HT1AR in HIP of ANH mice (taking in account the expression of GAPDH) looks to be higher as in CTRL and RES mice. The authors demonstrate, however, no differences. Less differences on the blot in 5HT1AR in P12 mice in HIP have been postulated as significant. Are there any explanations?

Figure S2: the number of animals per group should be provided.

© 2024. Published by The Company of Biologists under the terms of the Creative Commons Attribution License (https://creativecommons.org/licenses/by/4.0/).

Page 5 Paragraph 1: The section “For that, fixed slices from different brain regions (i.e., PFC, HP and RN) were stained with antibodies against 5-HT1AR and 5-HT7R, followed by the treatment with secondary antibodies attached to a unique short DNA strand. In case of interaction, DNA probes become located in a close proximity, which allows for rolling circle DNA amplification. Finally, amplified DNA was stained with a fluorescent detection reagent followed by fluorescence microscopy.” Belongs to the section “material and methods”.

Figure 4: “based on Allen brain atlas” should be “based on Allen brain atlas and shown as modified panels on the Figure”. As the captions of these panels are not readable, they should be removed, otherwise these panels should be enlarge / combined in one large one. Age of mice, which brain slices have been used for the analysis, should be provided.

Figure S3: Scale bar captions “10 um” is almost not readable and should be removed from the image.
Experimental mouse and age should be provided.

Figure 5: typo “immunohistochmical”. After “DAPI is shown in blue” a dot is missing. Scale bars in the figure legend are missing. The caption “10 um” is not readable and should be removed. Which color have PLA-positive blobs? Green? If they are green, how have authors separated them from serotonin staining?

Figure 6: The captions “10 um” on images are not readable, should be removed and provided in the figure legend. The number of animals per group should be provided.

Page 8 Paragraph 1 Line 6: number of heterodimers in PFC vs. HIP in CTRL group. If mentioned then it should be provided as a calculated number, not approximately.

4. Materials and methods

Page 13 Paragraph “Animals” Line 8: the authors should provide here the information, for which particular experiment the samples from mice from Hannover Medical School have been used.
Page 13 Paragraph “CUS” Line 3: italic formatting by [4,62] should be reset
Page 14 Paragraph “Social defeat stress” line 2: “mice” instead animals.
Page 15 Paragraph “Sucrose preference test” line 15: italic formatting by [30,63, 64] should be reset.
Page 16 Paragraph “qRT-PCR”: in general, the company and the cat numbers of used kits must be provided;
line 2 – the full name of the RLT Buffer should be provided.
Page 16 Paragraph “Western Blot” line 4: “20,000xg” instead “20.000xg”
Page 17 Paragraph “Western Blot” line 5: delete extra point “blot system. using”
Check the format for all numbers over 1000: 1.000 .. It should be the same format through the whole text.
Page 17 Paragraph “Immunohistochemistry” line 2: the authors should provide the information, how long and at which temperature the brains were postfixed. Line 5: “followed by methanol?” any solutions or steps in between? Cooling after 80 degree? It should be described. Furthermore, in this section, the catalogue numbers for all antibodies should be provided. “Primary antibodies were extensively washed”: the authors should provide an objective description.
Page 18 Paragraph “PLA”: the authors should provide the assay catalogue and company number, if self-prepared, descriptions of the buffers.
Page 19 Paragraph 2 “Analyze particles”: Whether the particles had a particular fluorophore spectra, or transferred into binary image, or nothing has been done and only the size has been used to identify the blobs it should be provided.
Page 20 Paragraph “Statistical analysis”: the authors should provide an explanation, in which analyses blinded analyses was not possible.

5. Discussion

Page 11 Paragraph 2: The postulation “Taken into consideration that expression levels of 5-HT1AR in PFC of adult mice is approximately six-fold higher than it for 5-HT7R, it means that selective increase in 5-HT7R expression obtained in PFC of RES animals will result in higher number of

© 2024. Published by The Company of Biologists under the terms of the Creative Commons Attribution License (https://creativecommons.org/licenses/by/4.0/).

heteromers, which can be implicated in the development of resiliency phenomenon" is, as far as not confirmed experimentally, in contradiction with the postulation "In our previous study, the highest affinity for complex formation was obtained for the 5-HT7R/5-HT7R homodimers, followed by the 5-HT7R/5-HT1AR heterodimers and 5-HT1AR/5-HT1AR homodimers".

First revision

Author response to reviewers' comments

Detailed response to the Reviewers´ comments

Reviewer 1

The experiments performed use sophisticatd methodology and multiple methods are used for detection. Certainly this increases validity of the data.

Reply:
We thank Reviewer for such positive evaluation.

Note grammatic errors, including p4 "Our study demonstrated that even though the expression levels ….; P9 'The situation differs in the RN, where twice as many 5-HT7R as 5-HT1AR were

Reply:
We have corrected all errors mentioned by Reviewer and performed additional proofreading of the revised manuscript.

Reviewer 2

Despite the high relevance of the topic, the tested hypothesis is not clear formulated, and the goal of the study is not defined. If the hypothesis is that: "The expression of the 5HT1AR is increased with aging and associated with depression, which can be attenuated by heteromerization with 5HT7R observed in resilient mice", then it must be stated very clear both, in abstract and in the introduction, and confirmed experimentally. Now, two stories to be observed: one is about receptors expression pattern during development and depression, another - about receptors heteromerization in depression. If the main hypothesis is that receptors heteromerization during development is an explanation for the resilience against stress in adult mice, then it should be clearly identified and confirmed experimentally.

Reply:
We thank Reviewer for pointing out this important aspect. We agree that clearly formulating the study's objective is crucial for general understanding. Therefore, in the revised manuscript we modified "Abstract" and "Introduction" sections in order to provide more clear statements. In particular, we highlighted that upon chronic stress amount of 5-HT1AR/5-HT7R heterodimers significantly reduces only in PFC of anhedonic mice, while it is not affected in resilient animals. This could be a consequence of increased 5-HT7R expression, which we obtained exclusively in the PFC of resilient animals, while no changes in 5-HT1AR expression between control and anhedonic mice were obtained. Taken together, our results suggest an important role of brain-region specific 5-HT1AR/5-HT7R heterodimerization for establishing depressive-like behavior and for development of resiliency.

An explanation should be provided in the section CUS model, why some offspring could become anhedonic and others resilient.

Reply:
We appreciate the Reviewer's suggestion. However, there is no definitive answer to this question yet. Multiple ongoing studies (including our own) are attempting to address this issue in details, in particular because of their enormous clinical impact. Numerous factors might influence the development of anhedonia or resilience. Despite a uniform genetic background and consistent stressors, differences in stress responses finally leading to anhedonia or resiliency can arise from

© 2024. Published by The Company of Biologists under the terms of the Creative Commons Attribution License (https://creativecommons.org/licenses/by/4.0/).

individual variations in epigenetics, posttranslational modification (e.g., palmitoylation), early life experiences, homeostatic mechanisms, and adaptive coping strategies. Since there is no clear answer explaining development of anhedonia and resilience, we prefer not to complicate a manuscript by discussing this issue, as it is not within the main scope of this study.

A graphical abstract would be very helpful to follow the process of proving of hypotheses with provided experimental design.

Reply:
We thank Reviewer for this suggestion. Since *Journal of Cell Science* does not accept graphical abstracts, we included it in the revised manuscript as a new Figure 7.

Minor Comments:

Abstract:
Page 2 Paragraph 2 Line 1 and 3: “mice” instead “animals” is clearer for the reader and simplify the search in data bases for mice studies. The authors should specify the animal species here. Page 2 Paragraph 2 Line 2: typo, should be „stages“ instead of „staged”

Reply:
We introduced changes suggested by Reviewer. In addition, we substantially revised “Abstract” section to better point out our main findings. We also adjusted the length of this section according to Author’s Guidelines.

Introduction
Page 4 Paragraph 2 Line 7-10: Here, the authors described only results in a CUS model, as a main finding. However, in the abstract they also described another finding - an increased expression of the 5HT1AR in three-months-old mice. This finding must be integrated also here with an interpretation/explanation of the results in a short form.

Reply:
We modified “Introduction” section according to the Reviewer’s comment.

Results
In general, the same order of the following list by defining analyzed brain regions through the manuscript: “prefrontal cortex, hippocampus and raphe nuclei” will be very helpful for understanding of the tested hypothesis, results and their interpretation.

Reply:
We unified the order of listing the analyzed brain regions to ensure consistency throughout the revised manuscript.

Figure 1, Panel a: The abbreviation “HP” should be the same as in the text: “HIP”, also in the figure legend and through the whole manuscript. ”HP” should be “HIP” as in the text. Figure 3 Panel a: The abbreviation “HP” should be the same as in the text: “HIP”, also in the figure legend.

Reply:
According to the Reviewer's suggestion, we used the abbreviation HP for hippocampus throughout the revised manuscript.

Figure 1. The number of WB experiments should be provided. Figure S1. The number of animals used for the analysis should be provided. Figure 2. The number of animals per each group should be provided. Figure 3 Panel a: The number of WB experiments should be provided. Figure S2: the number of animals per group should be provided. Figure 6: The number of animals per group should be provided.

Reply:
We would like to thank Reviewer for this important note. In the revised manuscript, we have added the number of WBs and animals to all relevant figures.
In general, for all figures: letters format should be the same on the panels and figure legend:

© 2024. Published by The Company of Biologists under the terms of the Creative Commons Attribution License (https://creativecommons.org/licenses/by/4.0/).

capital or not. This will simplify the reading.

Reply:
We have standardized the letter format in figure legends as well as in figures themselves using panel's labels with uppercase letters.

Page 5 Paragraph 2 Line 8 (expression ratios 5-HT1AR and 5-HT7R): The Figure 1D should be referred already in this sentence for a better understanding of the analysis.

Reply:
According to the Reviewer's suggestion, we referred to Figure 1D in the mentioned sentence.

Figure 1 Figure legend Panel (D): Typo, "ö" and "5-H7RR"
Reply:
In the revised version of the manuscript, we corrected all these typos.

Page 5 Paragraph 2: The authors supposed: "Taking into consideration our previous observation that the 5-HT7R possesses the highest affinity to form homodimers, followed by the 5-HT7R/5-HT1AR heterodimers and 5-HT1AR/5-HT1AR homodimers [17], our results suggest that the most of 5-HT7R expressed in PFC and HIP exist in form of heterodimers with the 5-HT1AR." This is contradictory, if the 5-HT7R possesses the highest affinity to form homodimers, why the most of 5-HT7R expressed in PFC and HIP should exist in form of heterodimers with the 5-HT1AR and not in homodimers? The authors must provide more convenient explanation.

Reply:
We apologize for not being sufficiently clear here. Above mentioned statement is based on results of our previous publication (*Renner et al., 2012, J Cell Sci*), where we developed a model to predict relative concentration of monomers and dimers at any given defined expression ratio between 5-HT1A and 5-HT7 receptors (see **Figure R1** below. This figure corresponds to the original Figure 3D in *Renner et al.*). According to this experimentally validated model, amount of 5-HT1AR/5-HT7R heterodimers at 10:1 ratio of 5-HT1AR to 5- HT7R (which was obtained in PFC at P90 and which correspond to the mol. fraction xD of 0.9) will be app. 6-fold higher then amount of 5-HT7R homodimers. In the revised manuscript, we provided more detailed explanation and hope that this part becomes clearer for the readers.

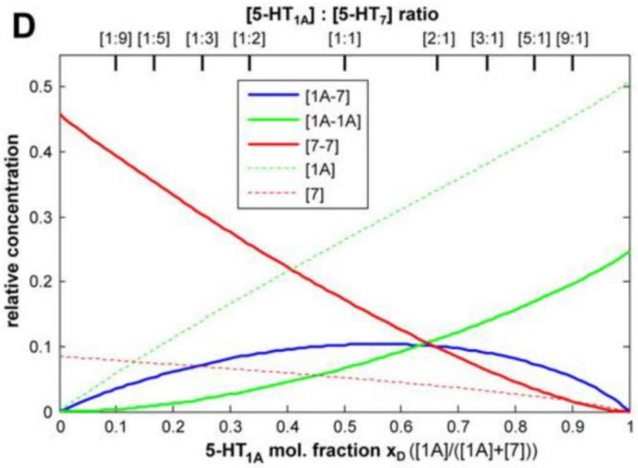

Figure R1. Relative concentrations of 5-HT1A and 5-HT7 homodimers (green and red solid lines), 5-HT1A/5-HT7 heterodimers (blue solid line) as well as of 5-HT1A and 5-HT7 monomers (green and red dashed lines) were calculated from values of dissociation constants and are shown as function of the donor mole fraction xD. The total concentration of receptors was assumed to be one. This figure was previously published as Fig. 3D in Renner et al., 2012 (doi: 10.1242/jcs.101337).

Page 5 Paragraph "Impact of Chronic Unpredictable Stress on 5-HT1A and 5-HT7R Expression Levels": if there no special reasons for capital letters in "Expression Levels" then it should be in

© 2024. Published by The Company of Biologists under the terms of the Creative Commons Attribution License (https://creativecommons.org/licenses/by/4.0/).

the same format as in the whole manuscript.

Reply:
We apologize for this oversight and have corrected it in the revised version of manuscript.

If the Figure 2a has been created by using some software such as BioRender, it should be mentioned in the figure legend. The abbreviations “SPT”, “W”, “FST” should be provided in the figure legend.

Reply:
In line with the Reviewer's suggestion, we added missing information about illustration software and abbreviations.

The quality of the WB of 5HT7R in PFC, HP and RN is very low and should be improved. Also, the expression of the 5HT1AR in HIP of ANH mice (taking in account the expression of GAPDH) looks to be higher as in CTRL and RES mice. The authors demonstrate, however, no differences. Less differences on the blot in 5HT1AR in P12 mice in HIP have been postulated as significant. Are there any explanations?

Reply:
We have included high-resolution images of representative WB in the revised version of manuscript. The quantitative analysis of WBs represents the average of multiple replicates, ensuring the robustness of our findings.
Regarding the expression of 5-HT1AR in the HP of ANH mice: While it may appear higher when considering the expression of GAPDH, statistical analysis shows no significant differences among the CTRL, ANH, and RES groups. In contrast, the differences in expression observed in the P12 mice in HP were statistically significant. **Table R1** shows raw intensity values as well as values obtained after sum of replicate analysis. We also included WB images for each repetition as an additional file “Blot transparency.pdf”. We hope that these combined data will provide a clearer view of the consistency and reproducibility of our results.

		**Raw intensity values**			**Ratio of raw intensity values**		**Sum of replicates and normalization CTRL to 1**	
		**5-HT1AR**	**5-HT7R**	**GAPDH**	**5-HT1AR/ GAPDH**	**5-HT7R/ GAPDH**	**5-HT1AR/ GAPDH**	**5-HT7R/ GAPDH**
HP	CTRL1	9234,238	4965,077	4735,589	1,950	1,048	1,038	1,083
HP	ANH1	12614,016	3908,999	6400,489	1,971	0,611	1,050	0,631
HP	RES1	12065,309	4298,734	7446,631	1,620	0,577	0,863	0,596
**HP**	**CTRL2**	**8412,530**	**3857,386**	**7856,388**	**1,071**	**0,491**	**0,861**	**0,955**
**HP**	**ANH2**	**11913,016**	**2967,909**	**7354,803**	**1,620**	**0,404**	**1,302**	**0,785**
**HP**	**RES2**	**8844,773**	**2647,707**	**9017,309**	**0,981**	**0,294**	**0,788**	**0,571**
HP	CTRL3	6511,631	2082,897	7929,874	0,821	0,263	1,101	0,962
HP	ANH3	5644,924	1744,993	8367,723	0,675	0,209	0,904	0,764
HP	RES3	6042,338	1364,442	8568,995	0,705	0,159	0,945	0,583

© 2024. Published by The Company of Biologists under the terms of the Creative Commons Attribution License (https://creativecommons.org/licenses/by/4.0/).

Table R1. Quantification of band intensities corresponding to 5-HT1AR, 5-HT7R, and GAPDH in the HP from the multiple WBs based on the sum of replicates approach. Analysis for WB images presented in Fig. 3A of the main manuscript is highlighted in red.

Page 5 Paragraph 1: The section "For that, fixed slices from different brain regions (i.e., PFC, HP and RN) were stained with antibodies against 5-HT1AR and 5-HT7R, followed by the treatment with secondary antibodies attached to a unique short DNA strand. In case of interaction, DNA probes become located in a close proximity, which allows for rolling circle DNA amplification. Finally, amplified DNA was stained with a fluorescent detection reagent followed by fluorescence microscopy." Belongs to the section "material and methods".

Reply:
We agree and removed the mentioned text from the 'Results' section. Part of that is now included into the 'Materials and Methods' section.

Figure 4: "based on Allen brain atlas" should be "based on Allen brain atlas and shown as modified panels on the Figure". As the captions of these panels are not readable, they should be removed, otherwise these panels should be enlarge / combined in one large one. Age of mice, which brain slices have been used for the analysis, should be provided.

Reply:
According to the Reviewer's suggestion, we replaced the schematic presentation of brain regions and changed the text accordingly. In addition, we added information about the age of animals to the figure legend.

Figure S3: Scale bar captions "10 um" is almost not readable and should be removed from the image. Experimental mouse and age should be provided.

Reply:
As suggested by Reviewer, we removed "10μm" caption. In the figure legend, we mentioned that experiments were performed using brain slicers from CUS control animals at P120.

Figure 5: typo "immunohistochmical". After "DAPI is shown in blue" a dot is missing. Scale bars in the figure legend are missing. The caption "10 um" is not readable and should be removed.

Reply:
We removed the "10μm" caption from the image and added the scale bar size to the figure legend.

Figure 5: Which color have PLA-positive blobs? Green? If they are green, how have authors separated them from serotonin staining?

Reply:
We apologize for not being clear enough at this point. In initial version, only serotonin staining (green) and nuclei (blue) were shown. In the revised version, we added a second inset showing the PLA signal in red, which was not shown before. In addition, we added extended PLA analyses part by explaining how the PLA-positive blobs were detected.

Figure 6: The captions "10 um" on images are not readable, should be removed and provided in the figure legend.

Reply:
We have changed the figure according to the Reviewer's suggestion.

Page 8 Paragraph 1 Line 6: number of heterodimers in PFC vs. HIP in CTRL group. If mentioned then it should be provided as a calculated number, not approximately.

Reply:
In line with the Reviewer's comment, we added this information in the revised manuscript as follows: "… when comparing only CTRL mice, we observed the highest number of heterodimers in the PFC (PFC 13.5 ± 1.9; HP 0.5 ± 0.1; RN 0.8 ± 0.2).

© 2024. Published by The Company of Biologists under the terms of the Creative Commons Attribution License (https://creativecommons.org/licenses/by/4.0/).

Materials and methods
Page 13 Paragraph “Animals” Line 8: the authors should provide here the information, for which particular experiment the samples from mice from Hannover Medical School have been used.
Page 13 Paragraph “CUS” Line 3: italic formatting by [4,62] should be reset Page 14 Paragraph “Social defeat stress” line 2: “mice” instead animals.
Page 15 Paragraph “Sucrose preference test” line 15: italic formatting by [30,63, 64] should be reset.
Page 16 Paragraph “qRT-PCR”: in general, the company and the cat numbers of used kits must be provided;
line 2 – the full name of the RLT Buffer should be provided.
Page 16 Paragraph “Western Blot” line 4: “20,000xg” instead “20.000xg”
Page 17 Paragraph “Western Blot” line 5: delete extra point “blot system. using”
Check the format for all numbers over 1000: 1.000 .. It should be the same format through the whole text.
Page 17 Paragraph “Immunohistochemistry” line 2: the authors should provide the information, how long and at which temperature the brains were postfixed.
Line 5: “followed by methanol?” any solutions or steps in between? Cooling after 80 degree? It should be described.
Furthermore, in this section, the catalogue numbers for all antibodies should be provided. “Primary antibodies were extensively washed”: the authors should provide an objective description.
Page 18 Paragraph “PLA”: the authors should provide the assay catalogue and company number, if self-prepared, descriptions of the buffers.
Page 19 Paragraph 2 “Analyze particles”: Whether the particles had a particular fluorophore spectra, or transferred into binary image, or nothing has been done and only the size has been used to identify the blobs, it should be provided.

Reply:
We thank Reviewer for these valuable notes. In the revised manuscript, we have addressed all these points accordingly.

Page 20 Paragraph “Statistical analysis”: the authors should provide an explanation, in which analyses blinded analyses was not possible.

Reply:
Western blot analyses were not conducted in a blind manner due to the necessity of arranging the samples on the gel in a specific order. We added this information to the revised version of the manuscript.

Discussion
Page 11 Paragraph 2: The postulation “Taken into consideration that expression levels of 5- HT1AR in PFC of adult mice is approximately six-fold higher than it for 5-HT7R, it means that selective increase in 5-HT7R expression obtained in PFC of RES animals will result in higher number of heteromers, which can be implicated in the development of resiliency phenomenon” is, as far as not confirmed experimentally, in contradiction with the postulation “In our previous study, the highest affinity for complex formation was obtaine d for the 5-HT7R/5-HT7R homodimers, followed by the 5-HT7R/5-HT1AR heterodimers and 5-HT1AR/5-HT1AR homodimers”.

Reply:
We apologize for not being clear enough at this point. Here, we have a similar explanation as provided above for **Page 5, Paragraph 2** note of Reviewers. According to our model predicting relative concentration of dimers at any given defined expression ratio between 5-HT1AR and 5-HT7R (**Figure R1**, *Renner et al., 2012 J Cell Sci*), increase in 5-HT7R expression will shift 5-HT1AR mol. fraction x_D from 0.9 – i.e. value corresponding to 10:1 ratio between 5-HT1AR and 5-HT7R obtained in the PFC of control animals - to lower values (Figure R1). This will lead to increase in relative amount of 5-HT1AR/5-HT7R heterodimers. It means that selective increase in 5-HT7R expression obtained in PFC of RES animals will result in higher number of heterodimers, which might be implicated in the development of resiliency phenomenon. In the revised manuscript, we modified corresponding part of “Discussion” section accordingly.

© 2024. Published by The Company of Biologists under the terms of the Creative Commons Attribution License (https://creativecommons.org/licenses/by/4.0/).

Second decision letter

MS ID#: JOCES/2024/262219

MS TITLE: The interplay of serotonin 5-HT1A and 5-HT7 receptors in chronic stress

AUTHORS: Monika Bijata, Alexander Wirth, Jakub Wlodarczyk, and Evgeni Ponimaskin

We have now reached a decision on the above manuscript.

To see the reviewers' reports and a copy of this decision letter, please go to: https://submit-jcs.biologists.org and click on the 'Manuscripts with Decisions' queue in the Author Area. (Corresponding author only has access to reviews.)

As you will see, the reviewers gave favourable reports, however, one of the reviewers raised some critical points requiring your attention and some changes
to your manuscript. I hope you will be able to carry these out because I would like to accept your paper, depending on further comments from reviewers.

I would be grateful if you would also provide how you have dealt with the points raised by the reviewers in the 'Response to Reviewers' box.

Reviewer 1

Advance summary and potential significance to field

THe authors have revised the document in a manner that meets all of my concerns.

Mark M Rasenick

Comments for the author

The only suggestion: "Fewer" is more grammatically correct than "less" in figure 7.

Reviewer 2

Advance summary and potential significance to field

The authors answered all my comments and questions except one. Please see in the section "Suggestions to authors".

Comments for the author

Please provide the information to the Comment:

An explanation should be provided in the section CUS model, why some offspring could become anhedonic and others resilient.

Reply Authors:
We appreciate the Reviewer's suggestion. However, there is no definitive answer to this question yet.
Multiple ongoing studies (including our own) are attempting to address this issue in details, in particular because of their enormous clinical impact. Numerous factors might influence the development of anhedonia or resilience. Despite a uniform genetic background and consistent stressors, differences in stress responses finally leading to anhedonia or resiliency can arise from

© 2024. Published by The Company of Biologists under the terms of the Creative Commons Attribution License (https://creativecommons.org/licenses/by/4.0/).

individual variations in epigenetics, posttranslational modification (e.g., palmitoylation), early life experiences, homeostatic mechanisms, and adaptive coping strategies. Since there is no clear answer explaining development of anhedonia and resilience, we prefer not to complicate a manuscript by discussing this issue, as it is not within the main scope of this study.

Comment Reviewer 2: As the authors are using this model to investigate the importance of brain-region specific 5-HT1AR/5-HT7R heterodimerization for establishing depressive-like behavior and for development of resiliency, they must mention their own observations and of the others, even briefly.

Second revision

Author response to reviewers' comments

Detailed response to the Reviewers´ comments

Reviewer 1
The only suggestion: "Fewer" is more grammatically correct than "less" in figure 7.
Reply:
According to Reviewer's suggestion, we have replaced "less" by "fewer" in figure 7

Reviewer 2
Initial Comment
An explanation should be provided in the section CUS model, why some offspring could become anhedonic and others resilient.

Initial reply:
We appreciate the Reviewer's suggestion. However, there is no definitive answer to this question yet. Multiple ongoing studies (including our own) are attempting to address this issue in details, in particular because of their enormous clinical impact. Numerous factors might influence the development of anhedonia or resilience. Despite a uniform genetic background and consistent stressors, differences in stress responses finally leading to anhedonia or resiliency can arise from individual variations in epigenetics, posttranslational modification (e.g., palmitoylation), early life experiences, homeostatic mechanisms, and adaptive coping strategies. Since there is no clear answer explaining development of anhedonia and resilience, we prefer not to complicate a manuscript by discussing this issue, as it is not within the main scope of this study.

Comment Reviewer 2:
As the authors are using this model to investigate the importance of brain-region specific 5-HT1AR/5-HT7R heterodimerization for establishing depressive-like behavior and for development of resiliency, they must mention their own observations and of the others, even briefly.

Reply:
As suggested by Reviewer, we extended "Discussion" section of the revised manuscript by including the part, in which we extensively discussed possible reasons for development of anhedonic and resilient phenotypes (lines 278 – 286). Reference list has been adjusted accordingly.

Third decision letter

MS ID#: JOCES/2024/262219

MS TITLE: The interplay of serotonin 5-HT1A and 5-HT7 receptors in chronic stress

AUTHORS: Monika Bijata, Alexander Wirth, Jakub Wlodarczyk, and Evgeni Ponimaskin

© 2024. Published by The Company of Biologists under the terms of the Creative Commons Attribution License (https://creativecommons.org/licenses/by/4.0/).

I am happy to tell you that your manuscript has been accepted for publication in Journal of Cell Science, pending standard publication integrity checks.

© 2024. Published by The Company of Biologists under the terms of the Creative Commons Attribution License (https://creativecommons.org/licenses/by/4.0/).
